# Prolonged use of finasteride-induced gonadal sex steroids alterations, DNA damage and menstrual bleeding in women

**DOI:** 10.1042/BSR20191434

**Published:** 2020-02-07

**Authors:** Gadah Albasher, May Bin-Jumah, Saleh Alfarraj, Fatimah Al-Otibi, Nouf K. Al-Sultan, Saud Alarifi, Saad Alkahtani, Nahed S. Alharthi, Wedad S. Al-Qahtani

**Affiliations:** 1 Department of Zoology, College of Science, King Saud University, Saudi Arabia; 2 Department of Biology, College of Science, Princess Nourah bint Abdulrahman University, Saudi Arabia; 3 Department of Botany and Microbiology, College of Science, King Saud University, Saudi Arabia; 4 Department of Forensic Biology, College of Forensic Sciences, Naif Arab University for Security Sciences, Saudi Arabia; 5 Medical laboratory Science Department, College of Applied Medical Science, Princess Sattam Bin Abdulaziz University, Saudi Arabia

**Keywords:** 5α-reductase, DNA damage, Finasteride, heavy menstrual bleeding, mRNA expression, women patients

## Abstract

The aim of the present study was to examine the effect of prolonged use of finasteride on serum levels of dihydrotestosterone (DHT), estradiol (E2), progesterone, testosterone and androstenedione in women during the menstrual period. Further, to screen and compare the 5α-reductase activities through the expression of *SRD5A1, SRD5A2* and *AR* gene and to determine the level of *VEGF, VKOR* and *SAA* gene expression and DNA damage. A total of 30 Saudi women aged between 25 and 35 years were enrolled in the study. The selected women were divided into two groups. The first group (*n* = 15) received 5 mg finasteride/day for prolonged period of one year and second group (*n* = 15) was taken as a healthy control. ELISA technique was used for measuring the serum levels of the targeted hormones, and Comet assay was used for checking the DNA integrity. Our findings revealed significant decrement of DHT, E2, progesterone and androstenedione levels and elevated levels of testosterone in group treated with daily oral doses of 5 mg finasteride/day compared with the control subjects. mRNA expression suggested that finasteride has concrete effects on the gene expression of the selected genes from the treated group in comparison with the control group. In addition, finasteride induced DNA damage, and heavy menstrual bleeding was noted in women treated with finasteride. In conclusion, the present findings revealed that finasteride has adverse health effects in women associated with gonadal sex steroids alterations, DNA damage and heavy menstrual bleeding with no consensus in the treatment of androgenetic alopecia in women.

## Introduction

Female androgenetic alopecia (hair loss) is known to be a common problem in females that is difficult to understand and treat. Female alopecia involves a reduction in vertex hair density with the front hairline being retained [1–2]. Messenger and Sinclair (1) and Price (2) reported that about 10 percent of pre-menopausal in women have some evidence of androgenetic alopecia and the incidence increases (50–75 percent) in women 65 years or older menopausal women.

Finasteride slows down the process of the type II 5α-reductase enzyme that converts testosterone to dihydrotestosterone (DHT). This is the androgen that is responsible for developing the male pattern hair loss in genetically predisposed men. Drake et al. [3] recorded that 1 mg/day of oral finasteride significantly reduced serum DHT levels by a median 71.4% and reduced scalp DHT levels by 64.1% in men and the male pattern hair loss was treated for 1 year. It was also reported that there was a corresponding 9.1% (median) increase in testosterone levels from baseline but the levels remained within the normal physiological range. It was observed that oral finasteride 0.2–5 mg/day given for 4–6 weeks reduced DHT levels by up to 65% in the scalp and the desired site of action of finasteride in men with male pattern hair loss. Oliveira-Soares et al. [4] reported that finasteride treatment did not have any effect on the serum luteinizing hormone or follicle-stimulating hormone responses to gonadotropin-releasing hormone stimulation in healthy people. In addition, finasteride appeared not have influence on the hypothalamic–pituitary–testicular axis and the drug did not alter serum prolactin, sex hormone-binding globulin, aldosterone or cortisol levels in healthy people. It was reported that the level of serum estradiol slightly increased, but the level of testosterone remained within the normal range. The ratio of testosterone to estradiol was the same. The volume of ejaculation or the other measures of testosterone-mediated semen production such as sperm motility, morphology and number were not affected when finasteride 1 mg/day was used. Robic et al. [5] also reported that the dose of finasteride used had no effect on the volume of prostate in healthy men who are less than or equal to 41 years. The dose caused slightly reduced the level of serum prostate-specific antigen. Čeponis et al. [6] and Isidori et al. [7] showed evidences to support that finasteride has no effect on lipid or bone metabolism.

**Figure 1:**
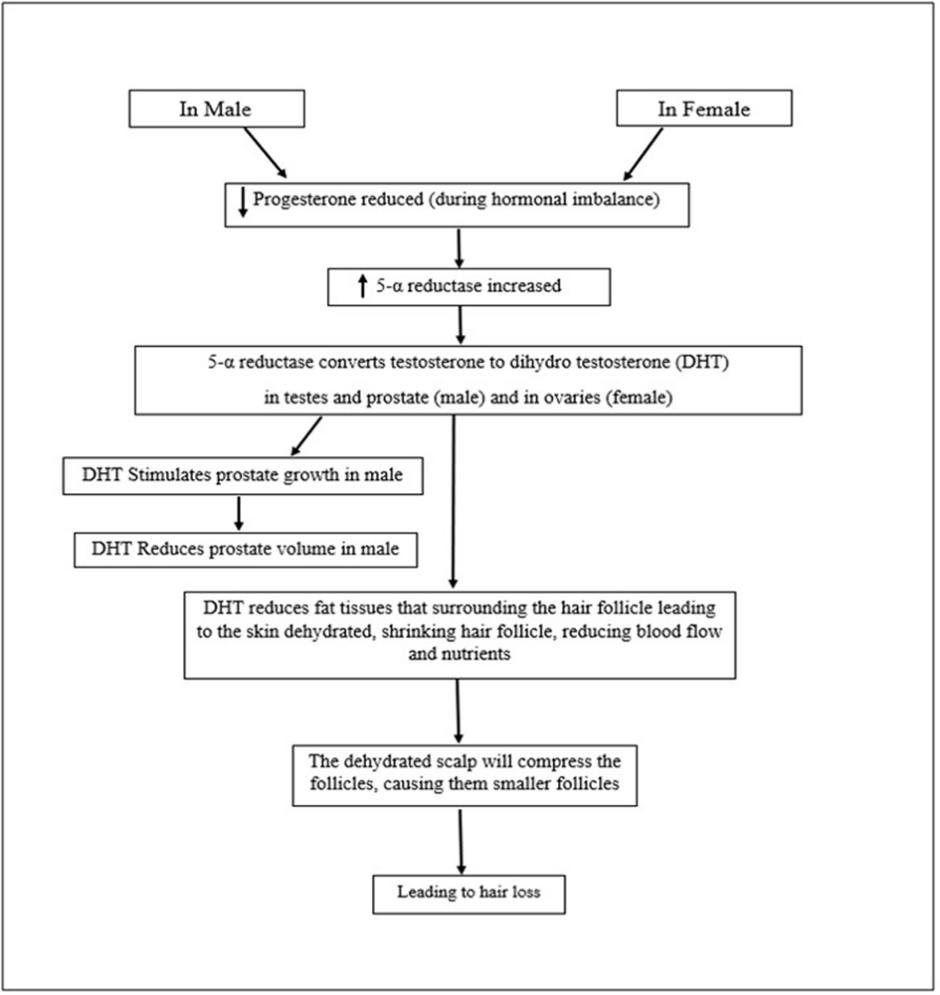
Flow diagram showing hair loss mechanism in male and female

Certain medications that act on the hormones of women may cause abnormal uterine bleeding through vagina and during normal menstrual period (Menorrhagia). Abebe [8] reported that these drugs or medications such as some herbal medications may interact with the mechanism of the steroid hormones and change the body’s level of hormones such as estrogen and progesterone. The main work of finasteride is to reduce the process in which 5-α reductase type II converts testosterone into DHT whereby there is a reduction fat tissues that surrounds the hair follicle that leads to the skin dehydrated, shrinking hair follicle, reducing blood flow and nutrients as shown in Figure 1. A DHT/AR complex is formed from a ligand-dependent transcription factor when DHT enters the cells and then selectively binds to the androgen receptor (AR). This complex is translocated into the ovaries and promotes the androgen response elements in women. Both isoforms of 5α-reductasethe I and II (SRD5A1 and SRD5A2) with AR gene play an important role in the androgen metabolism cascade. SRD5A2 has demonstrated with protection against PCOS and androgen disorder in women. Fluctuations in the activity of this enzyme (increased/decreased) in ovaries have been evaluated of affected women with endocrine dysfunction. The activity of the enzyme is influenced by different external and or internal factors that might be caused genetic alterations. It is therefore clear that the use of high concentration of hormonal drugs for a long period leads to irregular or heavy bleeding during the menstrual period. There is a balance between the estrogen and progesterone hormones in a normal menstrual cycle that regulates the building up of the lining of the uterus which eventually leads to bleeding during menstruation. The uterus produces excess of heavy flow of menstrual bleeding if there is hormonal imbalance. The SAA is the common serum biomarker of dysfunctional uterine bleeding. The properties and biological functions of angiogenic growth factors such as VEGF and VKOR have been demonstrated in several studies [9–12]. Testosterone, which is the main circulating androgen, must be converted into dihydrotestosterone by the 5α-reductase enzyme as shown in Figure 1 [13]. Messenger and Sinclair [1] and Robic et al. [5] record that most of the dihydrotestosterone in the prostate arise from testicular precursors while some may arise from adrenal precursors. Abebe [8], Baskind and Balen [14], Roberge et al. [15] and Traish et al. [16] also reported that the ovary and adrenal contribute to the intracellular dihydrotestosterone and the production of testosterone in women. The main objectives of this study are: (1) to determine the expression of *SRD5A1, SRD5A2 and AR* genes with *VEGF, VKOR* and *SAA* genes, (2) to evaluate the relationships between the targeted genes and the levels of biochemical and hormonal parameters in women patients that used 5 mg/day of finasteride, (3) to assess the effect of 5 mg/day of finasteride in women for prolong period on DNA integrity using Comet assay.

## Materials and methods

### Study subjects

A total of 30 Saudi women aged 25–35 years in Riyadh, Saudi Arabia were involved in the study. Healthy women have normal body weights were precipitated. The subjects were divided into two groups:

**Group I** – Women who did not use finasteride (normal control group) and taking no hormonal medications (*n* = 15).
**Group II** - Women who used oral daily doses (5 mg/day) of finasteride (positive control group) for prolonged period up one year and have also diagnosed with irregular and heavy menstrual bleeding (*n* = 15).

The Ethics Committee of Princess Nourah bint Abdulrahman University in Riyadh (Saudi Arabia) approved the present study (IRB long number: h-01-R-0659-2018-329), performed in accordance with the guidelines of the Declaration of Helsinki. Informed consents were obtained from all participated donors before entering the study.

### Sample collection and preparation

Blood samples (5 ml) were collected in the morning during the menstrual period at the second day on an empty stomach. The samples were mixed with anticoagulants, sterilized, and then allowed to stand for 1 h at room temperature. Afterward, the samples were centrifuged at 10,000 × ***g*** for 30 min at 4°C. Sera were collected, packaged and stored at −80°C for future use. Another set of 5 ml menstrual blood specimen were collected and mixed with anticoagulants, sterilized, and then allowed to stand for 20 min at room temperature. Afterward, the samples were centrifuged at 2000 × ***g*** for 30 min at 4°C. Sera were collected, and the supernatants were packaged and stored at −80°C until use. All the specimens used in the study were coded, and the patient’s confidentiality was preserved in accordance with the guidelines for studies of human subjects.

### RNA isolation and cDNA synthesis

RNA was extracted from the whole blood with TRIzol (Invitrogen) and total RNA was extracted according to the manufacturer’s instructions (Promega). Briefly, cells were lysed with guanidinium thiocyanate, followed by RNA extraction with acid phenol and chloroform–isoamyl alcohol (24:1). RNA, which was present in the top aqueous phase, was purified by adsorption to an RNA matrix. About 2 µg of total RNA was reversed into cDNA using QuantiTect Reverse Transcription kit (Qiagen, cat No: 205313), as a template for qPCR as described.

### RT-PCR

About 2 µg of cDNA was added as triplicate for each sample in the RT-PCR system to quantity the relative mRNA levels of β-actin, *SRD5A1, SRD5A2 AR, VEGF, VKOR* and *SAA* genes. PCR was performed using the primers shown in Table 1. The following PCR conditions were used: 95°C for 5 min, followed by 30 cycles at 95°C for 30 s, 56°C for 20 s, and 72°C for 40 s, and a final step of 72°C for 10 min. All qPCR reactions were performed in triplicate. Sequences of primers used are listed in Table 1.

**Table 1:** Sequences of target primers and associated genes in women

Gene symbol	Gene name/function	Forward primer	Reverse primer
β-Actin	β-Actin	5′-GGC ATC CAC GAA ACT ACC T-3′	5′-TCG TCC TCA TAC TGC TCA GG-3′
*SRD5A1*	Steroid 5 α-reductase 1	5′-GAA GCC TGA CTT GAG AAC CCT-3′	5′-AAA TTA AGC ACC GAT GCC CG-3′
*SRD5A2*	Steroid 5 α-reductase 2	5′-GAT GCA GGT TCA GTG CCA GC-3′	5′-CAG GGC ATA GCC GAT CCA TT-3′
*AR*	Androgen receptor	5′-CAT GTG GAA GCT GCA AGG TCT-3′	5′-GTG TAA GTT GCG GAA GCC AGG-3′
*VEGF*	Vascular endothelial growth factor A	5′-GGA GGC TGG CAA CAT AAC-3	5′-TGG CTC TTA AAC TAC TTT T-3′
*VKOR*	Making a vitamin K epoxide reductase	5′-GCA GAC TTC ATT TCC CTT-3	5′-TTT GGC TCA CTT GGC A-3
*SAA*	Serum amyloid A protein	5′-CTT GCC AGG TAG ACG C-3′	5′-TTT TCA GTT GGT CGC G-3′

### ELISA

The sera that were collected from different groups, DHT, E2, progesterone, testosterone and androstenedione were analyzed using ELISA kits according to manufacturer’s instructions.

### Determination of DNA strand breakage

A volume of 20 μl of whole blood was mixed with 1 ml RPMI 1640 in a microcentrifuge tube, to which 100 μl Ficoll histopaque was added below the blood/media mixture. The contents were centrifuged for 3 min at 2000 × ***g*** as described by Al-Gabely [17]. Thereafter, 100 μl of bottom of the media/top of Ficoll layer was removed and 1 ml media and contents were added, mixed and centrifuged for 3 min at 2000 × ***g*** to get a pellet of lymphocytes. The supernatant was poured off and the pellet was re-suspended in 75 μl of low melting point agarose (LMPA). The cells (1 × 10^7^ cells) were collected and equal volume of cell suspension (4 × 10^5^) was mixed with 0.7% (w/v) low melting agarose (LMA). The mixture was pipetted onto the frosted slides pre-coated with 1.0% (w/v) normal melting agarose.

After solidification of agarose, the slides were covered with another 100 ml of 0.7% (w/v) LMA and immersed in lysis buffer (2.5 M NaCl, 100 mM EDTA, 10 mM Tris–HCl buffer, 0.1% SDS and 1% Triton X-100 and 10% DMSO; pH 10.0) for 90 min to lyse the cellular and nuclear membranes. The slides were then washed twice with neutralizing buffer (0.4 M Tris–HCl; pH 7.5) for 10 min and treated with ethanol for another 5 min. The slides were stained with 40 ml of SYBR green (20 mg/ml) and DNA damage was visualized by using fluorescence microscope (Olympus, Japan’’ equipped with Cool SNAP1 Pro color digital camera).

The damage appeared as a ‘Comet’ with fragmented DNA (tail) being separated from undamaged nuclear DNA (head), and measurements were made by a Comet 5 image analysis software developed by Kinetic Imaging, Ltd. (Liverpool, U.K.) linked to a CCD camera to assess the quantitative and qualitative extent of DNA damage in the cells by measuring the tail length of DNA migration (µm) and the percentage of migrated DNA. Finally, the program calculates tail moment. Generally, 50–100 randomly selected cells were analyzed per sample.

### Statistical analysis

Statistical analysis was carried out by using two statistical approaches; two-way ANOVA approach was performed to check the effect of finasteride on DNA damage from the results that obtained by the Comet assay method and Tukey multiple comparisons test was performed to check the effect of finasteride on hormone levels using GraphPad Prism software (version 3.0 cx; GraphPad Inc., San Diego, CA). We have also used cluster analysis approach to show the correlation of finasteride effects on the hormone levels and the gene expression across both studied groups. Each experiment was repeated at least three times. Values were considered statistically significant at *P* < 0.05.

## Results

### Menstruation pattern

The majority of women in finasteride group noticed menorrhagia, also known as heavy menstrual bleeding (HMB) and/or metrorrhagia, a uterine bleeding at irregular intervals between the menstrual periods, with increased in days of period.

### Cluster analysis for sera steroid hormone levels in mid-luteal phase

The result gives *P*-values of 0.00, 0.00 and 0.00 for the hormones levels, category of use of finasteride (that is normal and use of finasteride) and an interaction effect. These values show that there are effects of using finasteride and there are also difference among the hormone levels and the women who used or did not use finasteride. For instance, the Tukey test was used to check for the actual hormone levels that differed. Tables 2 and 3 show the *P* values of the Tukey test that was used to check for the hormone levels and ratio, respectively, which differed.

**Table 2:** Table shows the effect of finasteride on the sera levels of targeted hormones from female group who used oral daily doses (5 mg/day) of finasteride

Hormones	Control patients	Finasteride group
**DHT (pg/ml)**	39.6 ± 9.29	8.4 ± 0.89*
**E2 (estradiol) (pg/ml)**	41.4 ± 8.85	11.2 ± 1.31*
**Progesterone (nmol/l)**	2.18 ± 0.24	0.22 ± 0.03*
**Testosterone (pg/ml)**	2.72 ± 0.36	8.8 ± 0.58*
**Androstenedione (ng/ml)**	2.1 ± 0.38	0.53 ± 0.06*

Values are expressed as means ± SD; *n* = 15 for each group. (*): significant difference compared to the control group.

**Table 3:** Table shows the effect of finasteride on *P*-values of the sera ratio of targeted hormones from female group who used oral daily doses (5 mg/day) of finasteride in comparison with the control group

Hormones	*P*-values
DHT-Androstenedione	0.20
E2-Androstenedione	0.00
Progesterone-Androstenedione	0.999
Testosterone-Androstenedione	0.999
E2-DHT	0.00
Progesterone-DHT	0.184
Testosterone-DHT	0.12
Progesterone-E2	0.00
Testosterone-E2	0.00
Testosterone-Progesterone	0.999

**Figure 2:**
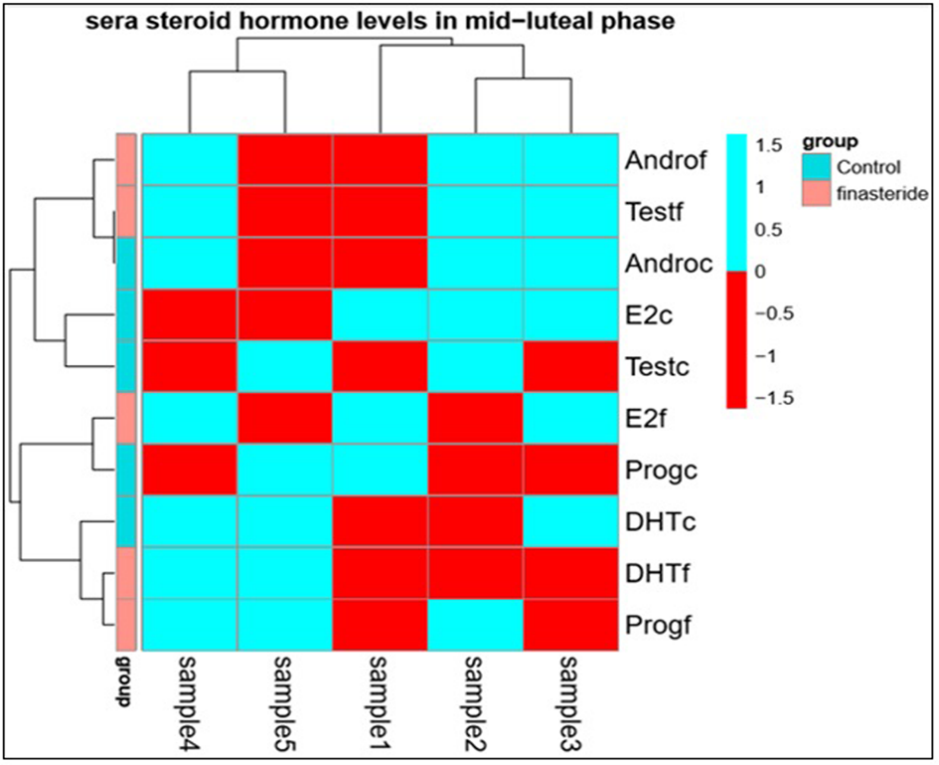
Figure shows three clusters in different colors with significant correlation among the hormone levels between both groups that were assigned into hierarchical clusters

We will note that *P*-values greater than 0.05 are taken to show differences in the hormone levels. Cluster analysis was also performed for the hormone levels of normal woman and women who had used finasteride. The aim was to see if there are clusters of women who had more similar patterns. We have used the “hierarchical clustering” to identify clusters as shown in Figure 2.

### DNA damage data by Comet assay

**Figure 3:**
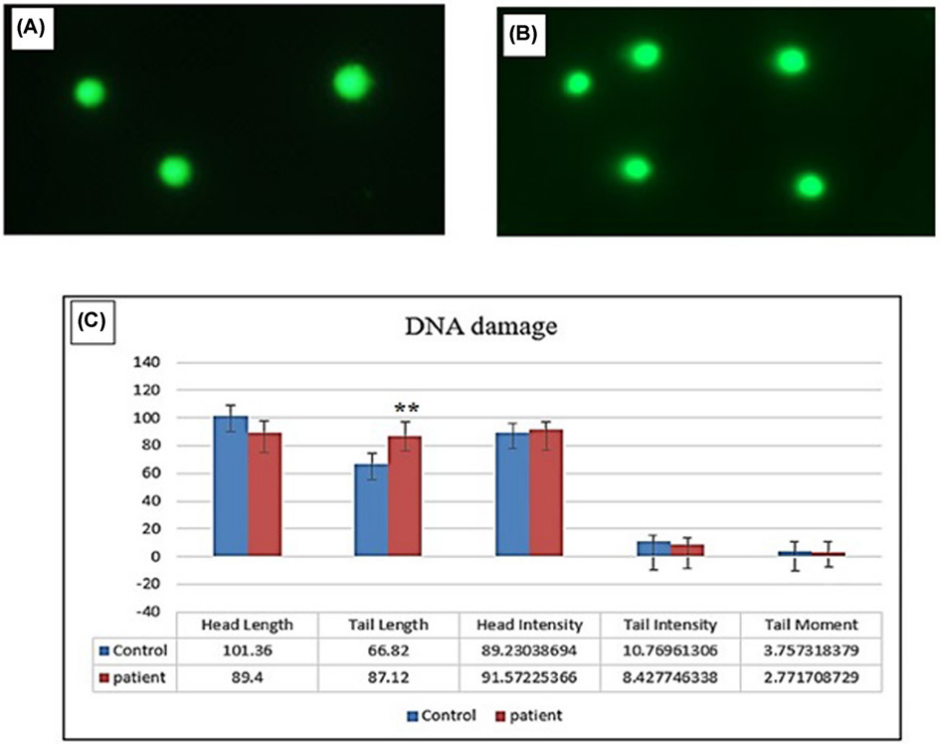
Finasteride-induced DNA damage in lymphocytes from female who used only oral daily doses (5 mg/day) of finasteride (**A**) Control (normal diet). (**B**) female who used oral daily doses (5 mg/day) of finasteride. (**C**) Showing quantified data of head DNA length, tail length, head intensity, tail intensity and moment. All values are represented as mean ± SE (*n* = 15). Differences were considered statistically significant at ** *P* < 0.01.

The Comet assay revealed that finasteride induced DNA damage in lymphocytes from female group who used oral daily doses (5 mg/day) of finasteride for prolonged period up one year compared with control groups (Figure 3). However, finasteride induced a slight DNA damage in lymphocytes in comparison with the control group.

Tail length from female group who used oral daily doses (5 mg/day) of finasteride was significant higher (*P* ≤ 0.01) as compared with the control group. On other hand, a non-significant fluctuation in the head-length of the DNA, head and tail intensity, length and moment of the DNA tail was observed in both groups.

### mRNA expression profiling of both sets of target selected genes

**Figure 4:**
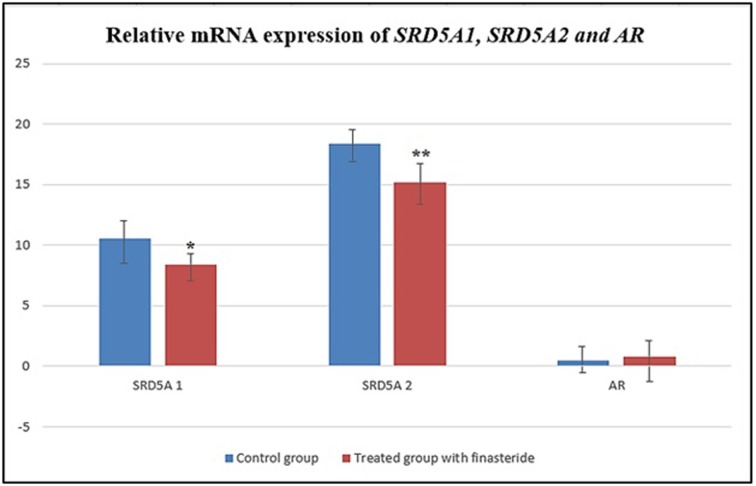
Relative mRNA expression for *SRD5A1, SRD5A 2* and *AR*, the gene expression was normalized using the most stable housekeeping gene *GAPDH* All values are represented as mean ± SE (*n* = 15). Differences were considered statistically significant at * *P* < 0.05 and ** *P* < 0.01.

The results on mRNA expression levels of *SRD5A1, SRD5A2 and AR* genes were recorded a significant decrease in *SRD5A1 and SRD5A2*, while *AR* gene expression revealed a significant increase with *P*-values of 0.000, 0.000 and 0.0004 respectively for the gene types, category of use of finasteride (which is control and use of finasteride) and an interaction effect. These values show that there are effects of using finasteride and there are also difference among the gene and the women who used or did not use finasteride. The Tukey test was used to check for the actual gen that differed. The *P*-values of 0.00, 0.00 and 0.00 show that the all gene types (*SRD5A1, SRD5A2* and *AR*) differed as shown in Figure 4.

**Figure 5:**
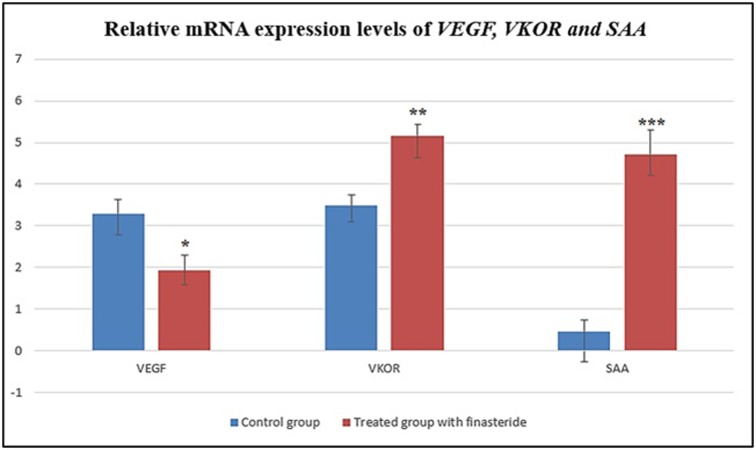
Relative mRNA expression for *VEGF, VKOR and SAA*, the gene expression was normalized using the most stable housekeeping gene *GAPDH* All values are represented as mean ± SE (*n* = 15). Differences were considered statistically significant at * *P* < 0.05, ** *P* < 0.01 and *** *P* < 0.001.

We have also used a two-way ANOVA to check whether there is effect of finasteride on gene (*VEGF, VKOR* and *SAA*). The result gives *P*-values of 0.000, 0.000 and 0.000 for the gene types, category of use of finasteride (which is control and use of finasteride) and an interaction effect. These values were showed that there are effects of using finasteride and there are also difference among the gene and the women who used or did not use finasteride, where *VKOR* and *VEGF* gene expression were showed markedly induction, in contrast *SAA* gene expression was recorded a significant increase in female who administrated finasteride treatment for prolonged period as compared with the control group. The Tukey test was used to check for the actual gene that differed. The *P*-values of 0.00, 0.00 and 0.00 show that the all gene types (*VEGF, VKOR and SAA*) differed as shown in Figure 5.

**Figure 6:**
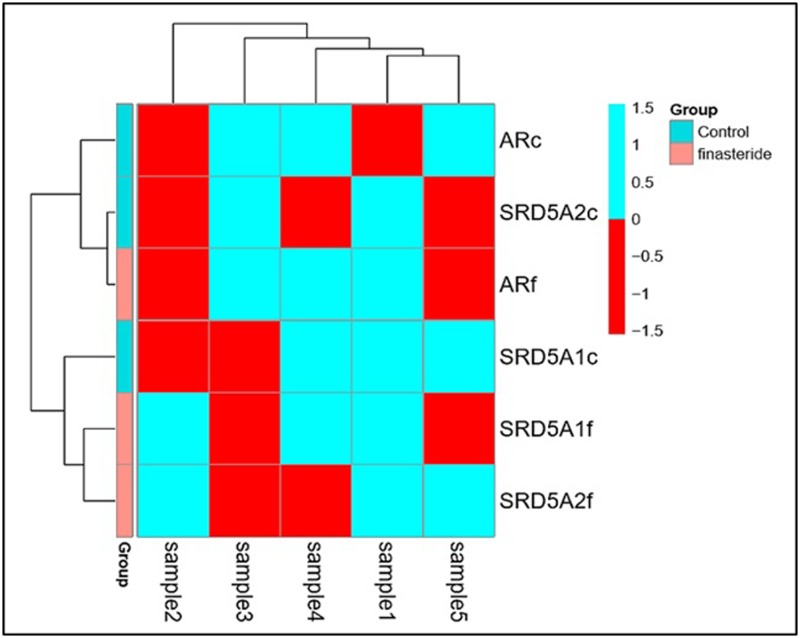
Figure shows three clusters in different colors with significant correlation among the mRNA expression of *SRD5A1, SRD5A2* and *AR* genes between both groups that were assigned into hierarchical clusters

**Figure 7:**
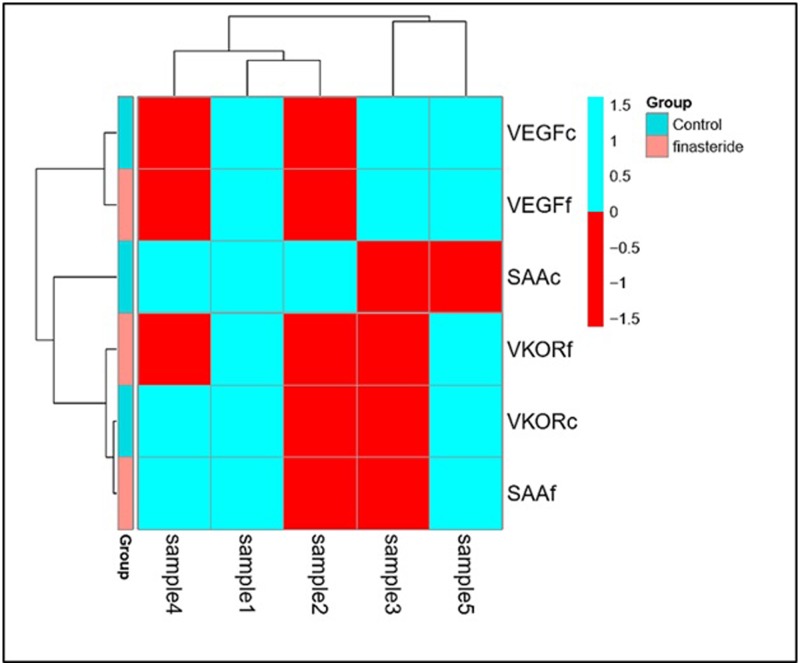
Figure shows three clusters in different colors with significant correlation among the mRNA expression of *VEGF, VKOR* and *SAA* genes between both groups which were assigned into clusters

Cluster analysis was performed to validate the clusters correlation that reflected finasteride effect on the mRNA expression levels of first set genes; SRD5A1, SRD5A2 and AR genes for the control and those who had used finasteride. As well as, Cluster analysis was applied on second set genes; VEGF, VKOR and SAA genes for the same aim and there were mix up of ‘‘hierarchical clustering” with all observations that were assigned into clusters on the studied groups as shown in Figures 6 and 7, respectively.

## Discussion

The aim of the present study was to validate whether the finasteride was associated with specific adverse effects like heavy menstrual bleeding, irregular menstrual cycles and abnormal levels in the steroid hormones such as DHT, E2, progesterone, testosterone and androstenedione in women who administrated finasteride for androgenetic alopecia treatment for prolonged period. Finasteride drug is known to be one of the inhibitor drugs used to treat people with prostatic hyperplasia, prostate cancer and pattern hair loss [18–19] but the effect of finasteride in women has not been well studied. In the present study, finasteride is seen to have caused DNA damage and menstrual bleeding in women who used finasteride (5 mg/day) as compared with women who did not use it. The enzyme 5-α reductase is produced in many male and female tissues like the reproductive tract, testes and ovaries. There are three isoenzymes of 5-α reductase; *SRD5A1, SRD5A2 and SRD5A3* [5,20].

The present study showed a significant decrease in the levels of DHT, E2 (estradiol), progesterone and androstenedione while in studies like Fruzzetti et al. [21], Kaufman et al. [19], Martyniuk et al. [20] and Moghetti et al. [22] recorded a marked increase in testosterone due to the effect of finasteride on 5-α reductase activity that prevents the conversion of testosterone to DHT. The present study has also used RT-PCR to demonstrate that vascular endometrial genes, functional gene clusters and upstream or downstream expression regulators are different among women who used finasteride with irregular menstrual cycles and heavy menstrual bleeding as compared with women have not used any hormonal medications. The mRNA expression showed increase in the *AR* levels and reduction in both *SRD5A1, SRD5A2*. This supports studies like Luo et al. [23], Stanbrough et al. [24] and Thomas et al. [25], which showed that the effect of finasteride inhibits 5-α reductase in order to treat hair loss in both men and women.

The present study also suggests that the mRNA expression of *VKOR* and *VEGF* were lower in the samples from female group who administrated finasteride as compared with the control group. It also suggested that *VKOR* and *VEGF* are associated with the irregular menstrual cycles and heavy menstrual bleeding (dysfunctional uterine bleeding) that confirms the findings by the study by Bao et al. [9].

Moreover, mRNA expression of SAA showed increase in women who used finasteride in comparison with the normal levels in control group. Thus, up-regulated expression of SAA levels could be a cause of uterine bleeding [9,11,12] and as a result of irregular uterine bleeding for prolonged periods [9,12]. The mRNA expression profiling was also validated using hierarchical clustering that revealed the effect of finasteride in women for long-term treatment.

Some studies showed complex changes of cellular and molecular mechanisms participating in the DNA damage, which are mediated by a diversity of abnormal regulation of aromatase [10,22,26–28]. Also, some other studies considered *SAA* gene as stress protein marker for DNA damage and cancer, which also support our findings on the DNA damage [10,28–30]. Furthermore, Alkahtane et al. [31] were recently found that finasteride administration to female mice has adverse effects within both the short and the long periods in female mice associated with apoptosis induction. Tu and Zini [32] also reported that low-dose finasteride may exert a harmful influence on sperm DNA integrity and sperm DNA injure in men, resulting in increased pregnancy losses.

In conclusion, the 5-α reductase inhibitor drug as finasteride is associated with common adverse health effects in women with long-term treatment for androgenetic alopecia such as; irregular menstrual cycle, aromatase disorder, high cholesterol, heavy menstrual bleeding and induced DNA damage. Long-term-treatment follow-up revealed that (5 mg/day) effects on hair growth are sustained in most women. Finally, the study recommended that finasteride should not be administrated as treatment for androgenetic alopecia in women.

## Study Boundaries

There are a number of limitations of the present study. First, the self-reported nature of the heavy menstrual bleeding could have resulted in inaccurate diagnosis. Second, due to the nature of Saudi Arabian cultural, women might feel uncomfortable discussing subject with their menstruation. Finally, the small number of patients can produce false-positive results. Further studies are required to address these limitations.

## Data Availability

All relevant data are within the paper.

## Abbreviations

DHT: dihydrotestosterone
LMA: low melting agarose
LMPA: low melting point agarose

## References

[1] Messenger A.G. and Sinclair R. (2006) Follicular miniaturization in female pattern hair loss: clinicopathological correlations. Br. J. Dermatol. 155, 926–930 10.1111/j.1365-2133.2006.07409.x 17034520

[2] Price V.H. (2003) Androgenetic Alopecia in Women. J. Investig. Dermatol. Symp. Proc. 8, 24–27, 2003/06/01/ 10.1046/j.1523-1747.2003.12168.x 12894991

[3] Drake L., Hordinsky M., Fiedler V., Swinehart J., Unger W.P., Cotterill P.C. et al. (1999) The effects of finasteride on scalp skin and serum androgen levels in men with androgenetic alopecia. J. Am. Acad. Dermatol. 41, 550–554 10495374

[4] Oliveira-Soares R., Maia e Silva J., Correia M.P. and Andre M.C. (2013) Finasteride 5 mg/day Treatment of Patterned Hair Loss in Normo-androgenetic Postmenopausal Women. Int. J. Trichol. 5, 22–25 10.4103/0974-7753.114709 23960392 PMC3746222

[5] Robic A., Feve K., Riquet J. and Prunier A. (2016) Transcript levels of genes implicated in steroidogenesis in the testes and fat tissue in relation to androstenone accumulation in fat of pubertal pigs. Domest. Anim. Endocrinol. 57, 1–9 10.1016/j.domaniend.2016.03.008 27285831

[6] Čeponis J., Wang C., Swerdloff R.S., Liu P.Y. (2017) Anabolic and metabolic effects of testosterone and other androgens: direct effects and role of testosterone metabolic products. Endocrinology of Testis and Male Reproduction. 1–22 Springer, Cham

[7] Isidori A.M., Balercia G., Calogero A.E., Corona G., Ferlin A., Francavilla S. et al. (2015) Outcomes of androgen replacement therapy in adult male hypogonadism: recommendations from the Italian society of endocrinology. J. Endocrinol. Invest. 38, 103–112 10.1007/s40618-014-0155-9 25384570 PMC4282686

[8] Abebe W. (2002) Herbal medication: potential for adverse interactions with analgesic drugs. J. Clin. Pharm. Ther. 27, 391–401 10.1046/j.1365-2710.2002.00444.x 12472978

[9] Bao S., Yang S.Y., Li Z.R. and Wen G.B. (2014) Comparison on serum biomarkers for anovulatory and ovulatory dysfunctional uterine bleeding in Lizu females. Asian Pac. J. Trop. Med. 7, 149–152 10.1016/S1995-7645(14)60012-2 24461530

[10] Marshak-Rothstein A. (2006) Toll-like receptors in systemic autoimmune disease. Nat. Rev. Immunol. 6, 823–835 10.1038/nri1957 17063184 PMC7097510

[11] Perry M.E., Stirling A. and Hunter J.A. (2008) Effect of etanercept on serum amyloid A protein (SAA) levels in patients with AA amyloidosis complicating inflammatory arthritis. Clin. Rheumatol. 27, 923–935 10.1007/s10067-008-0875-3 18379834

[12] Li G., Ren F., Yao J., Wang M., Feng X. and Liu D. (2013) Human serum amyloid A (SAA) protein changes in acute epilepsy patients. Int. J. Neurosci. 123, 265–278 10.3109/00207454.2012.756876 23230824

[13] Albasher G., Albrahim T., Alsultan N., Alfaraj S., Alharthi M.S., Kassab R.B. et al. (2019) Red beetroot extract mitigates chlorpyrifos-induced reprotoxicity associated with oxidative stress, inflammation, and apoptosis in rats. Environ. Sci. Pollut. Res. Int. 10.1007/s11356-019-07009-6 31823260

[14] Baskind N.E. and Balen A.H. (2016) Hypothalamic-pituitary, ovarian and adrenal contributions to polycystic ovary syndrome. Best Pract. Res. Clin. Obstet. Gynaecol. 37, 80–97 10.1016/j.bpobgyn.2016.03.005 27137106

[15] Roberge C., Carpentier A.C., Langlois M.F., Baillargeon J.P., Ardilouze J.L., Maheux P. et al. (2007) Adrenocortical dysregulation as a major player in insulin resistance and onset of obesity. Am. J. Physiol. Endocrinol. Metab. 293, E1465–E1478 10.1152/ajpendo.00516.2007 17911338

[16] Traish A.M., Vignozzi L., Simon J.A., Goldstein I. and Kim N.N. (2018) Role of Androgens in Female Genitourinary Tissue Structure and Function: Implications in the Genitourinary Syndrome of Menopause. Sex Med. Rev. 6, 558–571 10.1016/j.sxmr.2018.03.005 29631981

[17] Al-Gebaly A.S. (2017) Ameliorative Effect of Arctium lappa Against Cadmium Genotoxicity and Histopathology in Kidney of Wistar Rat. Pak. J. Biol. Sci. 20, 314–319 10.3923/pjbs.2017.314.319 29023056

[18] Hagberg K.W., Divan H.A., Persson R., Nickel J.C. and Jick S.S. (2016) Risk of erectile dysfunction associated with use of 5-alpha reductase inhibitors for benign prostatic hyperplasia or alopecia: population based studies using the Clinical Practice Research Datalink. BMJ 354, i4823 10.1136/bmj.i4823 27659058

[19] Kaufman K.D., Olsen E.A., Whiting D., Savin R., DeVillez R., Bergfeld W. et al. (1998) Finasteride in the treatment of men with androgenetic alopecia. Finasteride Male Pattern Hair Loss Study Group. J. Am. Acad. Dermatol. 39, 578–589 10.1016/S0190-9622(98)70007-6 9777765

[20] Martyniuk C.J., Bissegger S. and Langlois V.S. (2013) Current perspectives on the androgen 5 alpha-dihydrotestosterone (DHT) and 5 alpha-reductases in teleost fishes and amphibians. Gen. Comp. Endocrinol. 194, 264–274 10.1016/j.ygcen.2013.09.019 24095809

[21] Fruzzetti F., de Lorenzo D., Parrini D. and Ricci C. (1994) Effects of finasteride, a 5 alpha-reductase inhibitor, on circulating androgens and gonadotropin secretion in hirsute women. J. Clin. Endocrinol. Metab. 79, 831–835 8077369 10.1210/jcem.79.3.8077369

[22] Moghetti P., Castello R., Magnani C.M., Tosi F., Negri C., Armanini D. et al. (1994) Clinical and hormonal effects of the 5 alpha-reductase inhibitor finasteride in idiopathic hirsutism. J. Clin. Endocrinol. Metab. 79, 1115–1121 7962284 10.1210/jcem.79.4.7962284

[23] Luo J., Dunn T.A., Ewing C.M., Walsh P.C. and Isaacs W.B. (2003) Decreased gene expression of steroid 5 alpha-reductase 2 in human prostate cancer: implications for finasteride therapy of prostate carcinoma. Prostate 57, 134–139 10.1002/pros.10284 12949937

[24] Stanbrough M., Bubley G.J., Ross K., Golub T.R., Rubin M.A., Penning T.M. et al. (2006) Increased expression of genes converting adrenal androgens to testosterone in androgen-independent prostate cancer. Cancer Res. 66, 2815–2825 10.1158/0008-5472.CAN-05-4000 16510604

[25] Thomas L.N., Douglas R.C., Lazier C.B., Too C.K., Rittmaster R.S. and Tindall D.J. (2008) Type 1 and type 2 5alpha-reductase expression in the development and progression of prostate cancer. Eur. Urol. 53, 244–252 10.1016/j.eururo.2007.10.052 18006217

[26] Bulun S.E., Lin Z., Imir G., Amin S., Demura M., Yilmaz B. et al. (2005) Regulation of aromatase expression in estrogen-responsive breast and uterine disease: from bench to treatment. Pharmacol. Rev. 57, 359–383 10.1124/pr.57.3.6 16109840

[27] Schiewer M.J. and Knudsen K.E. (2016) Linking DNA Damage and Hormone Signaling Pathways in Cancer. Trends Endocrinol. Metab. 27, 216–225 10.1016/j.tem.2016.02.004 26944914 PMC4808434

[28] Lee Y.H., Sun Y., Gerweck L.E. and Glickman R.D. (2015) Regulation of DNA Damage Response by Estrogen Receptor beta-Mediated Inhibition of Breast Cancer Associated Gene 2. Biomedicines 3, 182–200 10.3390/biomedicines3020182 28536406 PMC5344223

[29] Ahamed M., Siddiqui M.A., Akhtar M.J., Ahmad I., Pant A.B. and Alhadlaq H.A. (2010) Genotoxic potential of copper oxide nanoparticles in human lung epithelial cells. Biochem. Biophys. Res. Commun. 396, 578–583 10.1016/j.bbrc.2010.04.156 20447378

[30] Pansarasa O., Bertorelli L., Vecchiet J., Felzani G. and Marzatico F. (1999) Age-dependent changes of antioxidant activities and markers of free radical damage in human skeletal muscle. Free Radical Biol. Med. 27, 617–622 10490283 10.1016/s0891-5849(99)00108-2

[31] Alkahtane A.A., Albasher G., Al-Sultan N.K., Alqahtani W.S., Alarifi S., Almeer R.S. et al. (2019) Long-term treatment with finasteride induces apoptosis and pathological changes in female mice. Hum. Exp. Toxicol. 38, 762–774 10.1177/0960327119842195 30943778

[32] Tu H.Y. and Zini A. (2011) Finasteride-induced secondary infertility associated with sperm DNA damage. Fertil. Steril. 95, 2125e13–2134e13 10.1016/j.fertnstert.2010.12.061 21292254

